# Publisher’s Note: We Changed Page Numbers to Article Numbers for Articles Published in *Infectious Disease Reports* Volume 1 to Volume 12, Issue s1

**DOI:** 10.3390/idr14020019

**Published:** 2022-02-22

**Authors:** Infectious Disease Reports Editorial Office

**Affiliations:** MDPI AG, St. Alban-Anlage 66, 4052 Basel, Switzerland; idr@mdpi.com

*Infectious Disease Reports* [1] was published by PAGEPress from Volume 1 (2009) to Volume 12, Issue s1 (2020). From Volume 12, Issue 3 (2020), *Gastroenterology Insights* is published by MDPI.

Previous articles in Volumes 1–12, Issue s1, published by PAGEPress in Open Access under a CC-BY (or CC-BY-NC-ND) licence, are now hosted by MDPI on mdpi.com as a courtesy and upon agreement with PAGEPress.

To standardize the metadata format of all previous articles, MDPI republished 250 articles in Volume 1 to Volume 12, Issue s1 with article numbers replacing page numbers (Table 1).

## Figures and Tables

**Table 1:** MDPI changed page numbers to articles numbers for 250 articles in Volume 1 to Volume 12, Issue s1.

DOI	Previous Page Number	Current Article Number
10.4081/idr.2009.1280	7–9	e4
10.4081/idr.2009.1285	1	e1
10.4081/idr.2009.1338	5–6	e3
10.4081/idr.2009.e2	2–4	e2
10.4081/idr.2009.e5	10–12	e5
10.4081/idr.2010.1281	9–10	e4
10.4081/idr.2010.1568	7–8	e3
10.4081/idr.2010.1585	40–46	e13
10.4081/idr.2010.1649	11–13	e5
10.4081/idr.2010.1723	14–15	e6
10.4081/idr.2010.1824	19–21	e8
10.4081/idr.2010.1939	47–48	e14
10.4081/idr.2010.1954	49–50	e15
10.4081/idr.2010.2138	51	e16
10.4081/idr.2010.e1	1–4	e1
10.4081/idr.2010.e2	5–6	e2
10.4081/idr.2010.e9	22–29	e9
10.4081/idr.2010.e10	30–32	e10
10.4081/idr.2010.e11	33–34	e11
10.4081/idr.2010.e12	35–39	e12
10.4081/idr.2010.e17	52–55	e17
10.4081/idr.2010.e18	56–63	e18
10.4081/idr.2011.1049	30–36	e7
10.4081/idr.2011.1794	3–6	e2
10.4081/idr.2011.2203	1–2	e1
10.4081/idr.2011.2260	62–63	e12
10.4081/idr.2011.2331	15–19	e5
10.4081/idr.2011.2349	12–14	e4
10.4081/idr.2011.2362	20–29	e6
10.4081/idr.2011.2370	52–61	e11
10.4081/idr.2011.2412	37–43	e8
10.4081/idr.2011.2440	44–45	e9
10.4081/idr.2011.3210	69–72	e15
10.4081/idr.2011.3257	77–82	e17
10.4081/idr.2011.3785	64–65	e13
10.4081/idr.2011.e3	7–11	e3
10.4081/idr.2011.e10	46–51	e10
10.4081/idr.2011.e14	66–68	e14
10.4081/idr.2011.e16	73–76	e16
10.4081/idr.2012.1935	1–2	e1
10.4081/idr.2012.3222	3–5	e2
10.4081/idr.2012.e3	6–8	e3
10.4081/idr.2012.e4	9–10	e4
10.4081/idr.2012.e5	11–13	e5
10.4081/idr.2012.e6	14–16	e6
10.4081/idr.2012.e7	17–18	e7
10.4081/idr.2012.e8	19–21	e8
10.4081/idr.2012.e9	22–29	e9
10.4081/idr.2012.e10	30–34	e10
10.4081/idr.2012.e11	35–37	e11
10.4081/idr.2012.e12	38–48	e12
10.4081/idr.2012.e13	49–51	e13
10.4081/idr.2012.e14	52–53	e14
10.4081/idr.2012.e15	54–55	e15
10.4081/idr.2012.e16	56–58	e16
10.4081/idr.2012.e17	59–62	e17
10.4081/idr.2012.e18	63–65	e18
10.4081/idr.2012.e19	66–70	e19
10.4081/idr.2012.e20	71–72	e20
10.4081/idr.2012.e21	73–76	e21
10.4081/idr.2012.e22	77–83	e22
10.4081/idr.2012.e23	84–89	e23
10.4081/idr.2012.e24	90–91	e24
10.4081/idr.2012.e25	92–100	e25
10.4081/idr.2012.e26	101–105	e26
10.4081/idr.2012.e27	106–113	e27
10.4081/idr.2012.e28	114–115	e28
10.4081/idr.2012.e29	116–119	e29
10.4081/idr.2012.e30	120–123	e30
10.4081/idr.2012.e31	124–125	e31
10.4081/idr.2012.e32	126–127	e32
10.4081/idr.2012.e33	128–133	e33
10.4081/idr.2012.e34	134–139	e34
10.4081/idr.2012.e35	140–141	e35
10.4081/idr.2012.e36	142–145	e36
10.4081/idr.2012.e37	146–151	e37
10.4081/idr.2013.e1	1–3	e1
10.4081/idr.2013.e2	4–5	e2
10.4081/idr.2013.e3	6–11	e3
10.4081/idr.2013.e4	12–15	e4
10.4081/idr.2013.e5	16–20	e5
10.4081/idr.2013.e6	21–23	e6
10.4081/idr.2013.e7	24–26	e7
10.4081/idr.2013.e8	27–31	e8
10.4081/idr.2013.e9	32–33	e9
10.4081/idr.2013.e10	34–36	e10
10.4081/idr.2013.e11	37–39	e11
10.4081/idr.2013.e12	40–42	e12
10.4081/idr.2013.e13	43–45	e13
10.4081/idr.2013.s1.e1	1	e1
10.4081/idr.2013.s1.e2	2–7	e2
10.4081/idr.2013.s1.e3	8–14	e3
10.4081/idr.2013.s1.e4	15–20	e4
10.4081/idr.2013.s1.e5	21–25	e5
10.4081/idr.2013.s1.e6	26–30	e6
10.4081/idr.2013.s1.e7	31–37	e7
10.4081/idr.2013.s1.e8	38–50	e8
10.4081/idr.2014.5050	1–4	5050
10.4081/idr.2014.5145	5–6	5145
10.4081/idr.2014.5147	17–19	5147
10.4081/idr.2014.5156	20–21	5156
10.4081/idr.2014.5157	7–8	5157
10.4081/idr.2014.5167	13–16	5167
10.4081/idr.2014.5327	9–12	5327
10.4081/idr.2014.5374	28–30	5374
10.4081/idr.2014.5387	24–27	5387
10.4081/idr.2014.5406	22–23	5406
10.4081/idr.2014.5413	38–40	5413
10.4081/idr.2014.5496	51–54	5496
10.4081/idr.2014.5497	31–33	5497
10.4081/idr.2014.5512	41–44	5512
10.4081/idr.2014.5513	34–37	5513
10.4081/idr.2014.5541	48–50	5541
10.4081/idr.2014.5576	55–61	5576
10.4081/idr.2014.5627	62–64	5627
10.4081/idr.2014.5646	45–47	5646
10.4081/idr.2015.5605	1–3	5605
10.4081/idr.2015.5726	7–9	5726
10.4081/idr.2015.5743	22–24	5743
10.4081/idr.2015.5747	4–6	5747
10.4081/idr.2015.5754	13–14	5754
10.4081/idr.2015.5774	15–18	5774
10.4081/idr.2015.5791	10–12	5791
10.4081/idr.2015.5801	32–34	5801
10.4081/idr.2015.5822	19–21	5822
10.4081/idr.2015.5849	25–27	5849
10.4081/idr.2015.5881	28–31	5881
10.4081/idr.2015.5901	46–49	5901
10.4081/idr.2015.5922	35–37	5922
10.4081/idr.2015.5937	42–43	5937
10.4081/idr.2015.5957	38–41	5957
10.4081/idr.2015.5967	66–73	5967
10.4081/idr.2015.5979	50–55	5979
10.4081/idr.2015.6031	60–65	6031
10.4081/idr.2015.6040	81–86	6040
10.4081/idr.2015.6103	44–45	6103
10.4081/idr.2015.6127	56–59	6127
10.4081/idr.2015.6173	74–76	6173
10.4081/idr.2015.6184	77–80	6184
10.4081/idr.2015.6286	91	6286
10.4081/idr.2015.6304	87–90	6304
10.4081/idr.2015.6356	92	6356
10.4081/idr.2016.5963	4–5	5963
10.4081/idr.2016.6213	1–3	6213
10.4081/idr.2016.6275	58–62	6275
10.4081/idr.2016.6320	6–7	6320
10.4081/idr.2016.6350	63–67	6350
10.4081/idr.2016.6368	8–11	6368
10.4081/idr.2016.6418	12–23	6418
10.4081/idr.2016.6494	68–70	6494
10.4081/idr.2016.6515	71–73	6515
10.4081/idr.2016.6545	78–79	6545
10.4081/idr.2016.6567	38–42	6567
10.4081/idr.2016.6568	24–32	6568
10.4081/idr.2016.6569	43–49	6569
10.4081/idr.2016.6570	33–37	6570
10.4081/idr.2016.6594	50–56	6594
10.4081/idr.2016.6612	74–75	6612
10.4081/idr.2016.6644	57	6644
10.4081/idr.2016.6817	80–82	6817
10.4081/idr.2016.6844	76–77	6844
10.4081/idr.2016.6950	83–84	6950
10.4081/idr.2017.6800	42–47	6800
10.4081/idr.2017.6821	13–17	6821
10.4081/idr.2017.6829	36–41	6829
10.4081/idr.2017.6836	28–31	6836
10.4081/idr.2017.6839	18–27	6839
10.4081/idr.2017.6849	8–12	6849
10.4081/idr.2017.6851	32–34	6851
10.4081/idr.2017.6884	58–61	6884
10.4081/idr.2017.6894	53–57	6894
10.4081/idr.2017.6900	62–65	6900
10.4081/idr.2017.6917	1–7	6917
10.4081/idr.2017.6930	73–76	6930
10.4081/idr.2017.7008	66–68	7008
10.4081/idr.2017.7017	50–52	7017
10.4081/idr.2017.7104	69–72	7104
10.4081/idr.2017.7142	91–93	7142
10.4081/idr.2017.7158	48–49	7158
10.4081/idr.2017.7185	87–90	7185
10.4081/idr.2017.7265	81–86	7265
10.4081/idr.2017.7268	77–80	7268
10.4081/idr.2018.7310	1–4	7310
10.4081/idr.2018.7409	9–12	7409
10.4081/idr.2018.7410	5–8	7410
10.4081/idr.2018.7522	16–18	7522
10.4081/idr.2018.7621	19–23	7621
10.4081/idr.2018.7632	26–27	7632
10.4081/idr.2018.7651	24–25	7651
10.4081/idr.2018.7654	42–44	7654
10.4081/idr.2018.7725	53–55	7725
10.4081/idr.2018.7731	39–41	7731
10.4081/idr.2018.7744	56–59	7744
10.4081/idr.2018.7746	60–63	7746
10.4081/idr.2018.7750	36–38	7750
10.4081/idr.2018.7765	30–35	7765
10.4081/idr.2018.7770	47–49	7770
10.4081/idr.2018.7773	45–46	7773
10.4081/idr.2018.7785	50–52	7785
10.4081/idr.2018.7804	64–66	7804
10.4081/idr.2019.7701	16–21	7701
10.4081/idr.2019.7872	1–3	7872
10.4081/idr.2019.7925	10–15	7925
10.4081/idr.2019.7975	4–9	7975
10.4081/idr.2019.8108	26–29	8108
10.4081/idr.2019.8132	22–25	8132
10.4081/idr.2019.8208	30–36	8208
10.4081/idr.2019.8272	37–40	8272
10.4081/idr.2020.8139	10–15	8139
10.4081/idr.2020.8304	19–21	8304
10.4081/idr.2020.8376	16–18	8376
10.4081/idr.2020.8510	28–31	8510
10.4081/idr.2020.8516	1–2	8516
10.4081/idr.2020.8523	32–34	8523
10.4081/idr.2020.8543	3–9	8543
10.4081/idr.2020.8609	25–27	8609
10.4081/idr.2020.8647	22–23	8647
10.4081/idr.2020.8716	1–4	8716
10.4081/idr.2020.8717	5–10	8717
10.4081/idr.2020.8718	11–13	8718
10.4081/idr.2020.8719	14–16	8719
10.4081/idr.2020.8720	17–20	8720
10.4081/idr.2020.8721	21–25	8721
10.4081/idr.2020.8722	26–28	8722
10.4081/idr.2020.8723	29–32	8723
10.4081/idr.2020.8724	33–35	8724
10.4081/idr.2020.8725	36–39	8725
10.4081/idr.2020.8726	40–43	8726
10.4081/idr.2020.8727	44–47	8727
10.4081/idr.2020.8728	48–50	8728
10.4081/idr.2020.8730	51–55	8730
10.4081/idr.2020.8731	56–60	8731
10.4081/idr.2020.8733	61–64	8733
10.4081/idr.2020.8734	65–67	8734
10.4081/idr.2020.8736	68–74	8736
10.4081/idr.2020.8737	75–77	8737
10.4081/idr.2020.8738	78–82	8738
10.4081/idr.2020.8740	83–87	8740
10.4081/idr.2020.8743	88–92	8743
10.4081/idr.2020.8744	93–97	8744
10.4081/idr.2020.8745	98–100	8745
10.4081/idr.2020.8746	101–104	8746
10.4081/idr.2020.8747	105–108	8747
10.4081/idr.2020.8748	109–112	8748
10.4081/idr.2020.8760	113–116	8760
10.4081/idr.2020.8761	117–120	8761
10.4081/idr.2020.8762	121–124	8762
10.4081/idr.2020.8763	125–127	8763
10.4081/idr.2020.8765	128–131	8765
10.4081/idr.2020.8766	132–136	8766
